# Distribution of molybdenum in soft tissues and blood of rats after intratracheal instillation of molybdenum(IV) sulfide nano- and microparticles

**DOI:** 10.1007/s43188-023-00213-0

**Published:** 2023-11-14

**Authors:** Renata Kuraś, Maciej Stępnik, Jarosław Grobelny, Emilia Tomaszewska, Magdalena Stanisławska, Katarzyna Domeradzka-Gajda, Wojciech Wąsowicz, Beata Janasik

**Affiliations:** 1 Central Laboratory, Nofer Institute of Occupational Medicine, 8 Teresy St., 91-348 Łódź, Poland; 2 QSAR LAB Ltd, 3 Lipy St., 80-172 Gdańsk, Poland; 3 Department of Toxicology and Carcinogenesis, Nofer Institute of Occupational Medicine, 8 Teresy St., 91-348 Łódź, Poland; 4 Department of Materials Technology and Chemistry, Faculty of Chemistry, University of Łódź, 163 Pomorska St., 90-236 Łódź, Poland; 5 Professor Emeritus, Nofer Institute of Occupational Medicine, 8 Teresy St., 91-348 Łódź, Poland; 6 Department of Chemical Safety, Nofer Institute of Occupational Medicine, 8 Teresy St., 91-348 Łódź, Poland

**Keywords:** Nanoparticles, Microparticles, Molybdenum(iv) disulfide, Bioimaging, Intratracheal instillation, Rat tissues

## Abstract

There is still little literature data on the toxicity and safety of the commonly used molybdenum (Mo) disulfide which is present in the working as well as living environments. Thus, an experiment was carried out involving rats, with single and repeated intratracheal exposure (in the latter case, 7 administrations at 2-week intervals with the analysis performed after 90 days) to lower (1.5 mg Mo kg^−1^ b.w.) and higher (5 mg Mo kg^−1^ b.w.) doses of molybdenum(IV) sulfide nanoparticles (MoS_2_-NPs) and microparticles (MoS_2_-MPs). The analysis of Mo concentrations in the tail and heart blood as well as in soft tissues (lung, liver, spleen, brain), after mineralization and bioimaging, was meant to facilitate an assessment of its accumulation and potential effects on the body following short- and long-term exposure. The multi-compartment model with an exponential curve of Mo concentration over time with different half-lives for the distribution and elimination phases of MoS_2_-MPs and MoS_2_-NPs was observed. After 24 h of exposure, a slight increase in Mo concentration in blood was observed. Next, Mo concentration indicated a decrease in blood concentration from 24 h to day 14 (the Mo concentration before the second administration), below the pre-exposure concentration. The next phase was linear, less abrupt and practically flat, but with an increasing trend towards the end of the experiment. Significantly higher Mo concentrations in MoS_2_-NPs and MoS_2_-MPs was found in the lungs of repeatedly exposed rats compared to those exposed to a single dose. The analysis of Mo content in the liver and the spleen tissue showed a slightly higher concentration for MoS_2_-NPs compared to MoS_2_-MPs. The results for the brain were below the calculated detection limit. Results were consistent with results obtained by bioimaging technique.

## Introduction

Currently, more and more attention is devoted to almost all areas of life of nanoparticles (NPs) and nanostructures sized 1‒100 nm. The use of metallic NPs is wide. Molybdenum disulphide IV (MoS_2_) is exerting an increasing impact on industrial applications. It is a transition metal dichalcogenide, an inorganic chemical compound in the IV oxidation state. The crystalline structure of MoS_2_ is a hexagonal layer of Mo atoms and 2 external hexagonal layers of chalcogen sulphur (S) atoms. The unique tribological properties of MoS_2_, resulting from its hexagonal crystal structure, make it widely used as a high-temperature lubricant, in dry, solid and liquid forms [1–3]. In a form of powder, MoS_2_ improves the parameters and durability of motorcycle and car engines. It is also used as a semiconductor and catalyst in the fuel and petrochemical industries. Physicochemical properties of Mo make it a useful alloy metal, both in the production of special steels as well as non-ferrous alloys and pigments. It is also used in the aerospace and defense industries, and in the production of Mo wires and rods for electric bulbs and furnaces [4, 5]. In accordance with Regulation (EC) No. 1272/2008, MoS_2_ is classified as a hazardous substance; H332—Harmful if inhaled [6, 7].

It is commonly known that the toxic properties of chemical substances and their adverse health effects in both occupationally and environmentally exposed populations may differ depending on the dose, time or route of exposure. The state of aggregation and the chemical form of a given metal are other factors affecting its toxic properties. Numerous animal studies have confirmed that NPs relatively quickly and easily overcome all the protective barriers of the body [8–11]. Works by Tjälve et al. [12], Oberdörster et al. [13], as well as Engin and Engin [14] have indicated that nanometer size particles reach the brain via the olfactory nerve. Therefore, they may come into contact with olfactory neurons in the olfactory epithelium, and be transported through olfactory cell axons to the olfactory bulb, where they directly affect the central nervous system [13]. However, little data is available on the distribution of MoS_2_-nanoparticles (MoS_2_-NPs) after inhalation or intratracheal exposure [15–17]. Uncertainty related to the safety and assessment of exposure to MoS_2_-NPs and/or MoS_2_-microparticles (MoS_2_-MPs) during their industrial production as well as during work in exposure to MoS_2_ results from insufficient knowledge on the mechanism of their toxicity.

The main objective of the study was the combination of advanced and specialized analytical methodologies [inductively coupled plasma mass spectrometry (ICP-MS), inductively coupled plasma optical emission spectrometry (ICP-OES), and laser ablation technique combined with inductively coupled plasma mass spectrometry with ionization of the sample (LA-ICP-MS)] allowing for the determination of MoS_2_-NPs and MoS_2_-MPs concentration in the blood, and mineralized tissue samples, as well as their spatial distribution and accumulation content in selected tissue sections (brain, lung, liver, spleen) obtained from the rats exposed to nano- and micrometer Mo particles, either once or 7 times at a dose of 1.5 mg Mo kg^−1^ b.w. and 5 mg Mo kg^−1^ b.w. (at 2-week intervals), or to polyvinylpyrrolidone (PVP) as a control substance, respectively, for each form into the trachea.

Moreover, MoS_2_-NPs as well as MoS_2_-MPs were used in an intratracheal instillation study in rats, in a single and repeated exposure model at doses of 1.5 and 5 mg MoS_2_ per kg b.w., to obtain data on the absorption and kinetics of MoS_2_ particles, including their possible accumulation in the body. It was hypothesized that biodistribution is dependent on the primary particle size, assessed distribution, and tissue accumulation at various time points, both during and after exposure. The possible implementation of this type of analysis in toxicological research was also assessed.

## Materials and methods

### Study design

Having been approved by the Ethics Committee for Animal Experiments (Resolution No. 6/ŁB 86/2018), the experimental study was performed using albino Wistar rats. The animals were at 6‒8 weeks of age and weighed 80‒120 g. The rats were acclimated for a week under 12-h day/12-h night cycles with unlimited access to water, at standard air humidity conditions as well as in temperature of 22 ± 3 °C. The general study design, including exposure time, the doses of Mo and the number of animals, was adopted in line with the guidelines reported by Warheit et al. [18, 19] and Ma-Hock et al. [20]. For further assay, at least 4 animals were selected, according to the OECD TG 417 Toxicokinetics guidelines (Adopted: 22 July 2010). Finally, a decision was made to follow the experimental scheme summarized in Table 1, assuming the exposure of animals to a single dose (1.5 or 5 mg MoS_2_ kg^−1^ b.w.) with the analysis performed after 24 h and 7 days, and a multiple dose (7 administrations at 2-week intervals).

**Table 1:** Study design including Mo doses, day of analysis, sampling and data collection

	Type of exposure/dose	Analysis	Tissue				
			Blood	Lung	Liver	Spleen	Brain
1	CON	7 days	–	+	+	+	+
2	1 administration 1.5 mg MoS_2_-NPs kg^−1^ b.w	24 h	Tail vein*	−	−	−	−
		7 days		+	+	+	+
3	1 administration 1.5 mg MoS_2_-MPs kg^−1^ b.w	24 h	Tail vein*	−	−	−	−
		7 days		+	+	+	+
4	1 administration 5 mg MoS_2_-NPs kg^−1^ b.w	24 h	Tail vein*	−	−	−	−
		7 days		+	+	+	+
5	1 administration 5 mg MoS_2_-MPs kg^−1^ b.w	24 h	Tail vein*	−	−	−	−
		7 days		+	+	+	+
6	CON	After 90 days	Venous blood from the heart during autopsy	+	+	+	+
7	7 administrations 1.5 mg MoS_2_-NPs kg^−1^ b.w	After 90 days	Tail vein*	−	−	−	−
8	7 administrations 1.5 mg MoS_2_-MPs kg^−1^ b.w	After 90 days	Tail vein*	−	−	−	−
9	7 administrations 5 mg MoS_2_-NPs kg^−1^ b.w	After 90 days	Tail vein* Venous blood from the heart during autopsy	+	+	+	+
10	7 administrations 5 mg MoS_2_sMPs kg^−1^ b.w	After 90 days	Tail vein* Venous blood from the heart during autopsy	+	+	+	+

*The blood was collected from the same animal before and after exposure (the animal was self-controlled)

### Reagents and standards

Multi-element CRM Comprehensive Mix B Standard 10.00 ± 0.05 mg per l (LGC, USA), ultra-pure deionized water from a Milli-Q water purification system (Millipore, Milli-Q Ellix 3, resistivity of 18.2 MΩ cm^−1^) and 65% nitric acid (HNO_3_, ULTREX II Ultrapure Reagent, J.T.Baker™), Triton-X (Sigma Aldrich) were used for the preparation of calibration standard solutions. Laboratory solid standards of agarose powder matrix (Sigma Aldrich, Darmstadt, Germany) were prepared as agarose gel tablets in the range of 0.5‒50 μg g^−1^ for the calibration by LA-ICP-MS.

Additionally, the ICP-MS method and the analytical procedure were verified by applying the available reference material Seronorm™ Trace Elements Whole Blood (Sero, Norway), as well as certified reference materials of the dogfish liver (DOLT-5, NRC-CNRC, Canada). Tablets of lyophilized reference material DOLT-5 using manual hydraulic press (Specac Atlas*™* Manual 15 T) were used to check the accuracy of LA-ICP-MS.

### ***Preparation of a colloid and a suspension of MoS***_***2***_

**Fig. 1:**
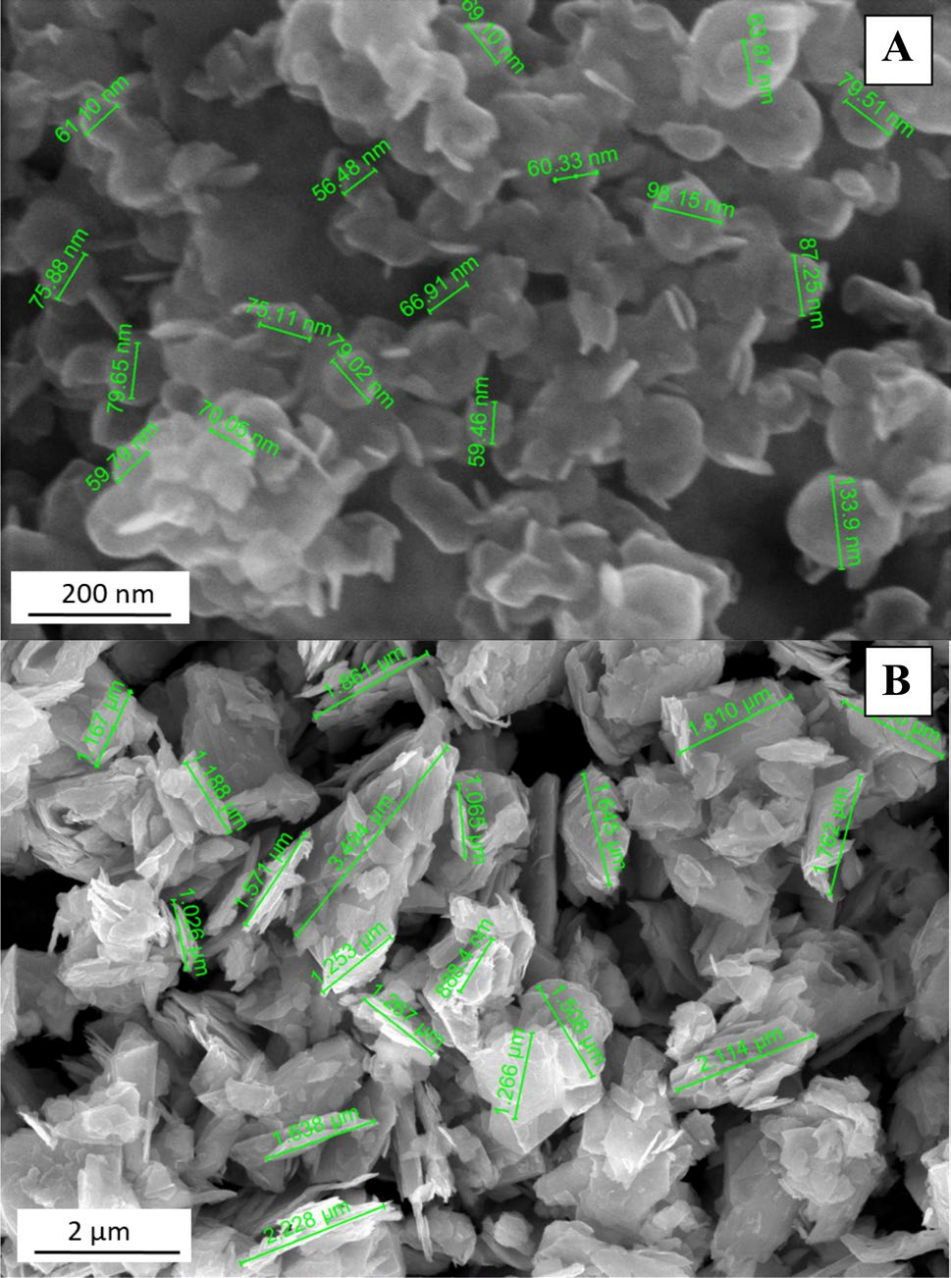
High-resolution scanning electron microscopy (FEI-Nova NanoSEM 450) images of MoS_2_-NPs (**a**) and MoS_2_-MPs (**b**).The scale bar is 200 nm and 2 µm, respectively

**Fig. 2:**
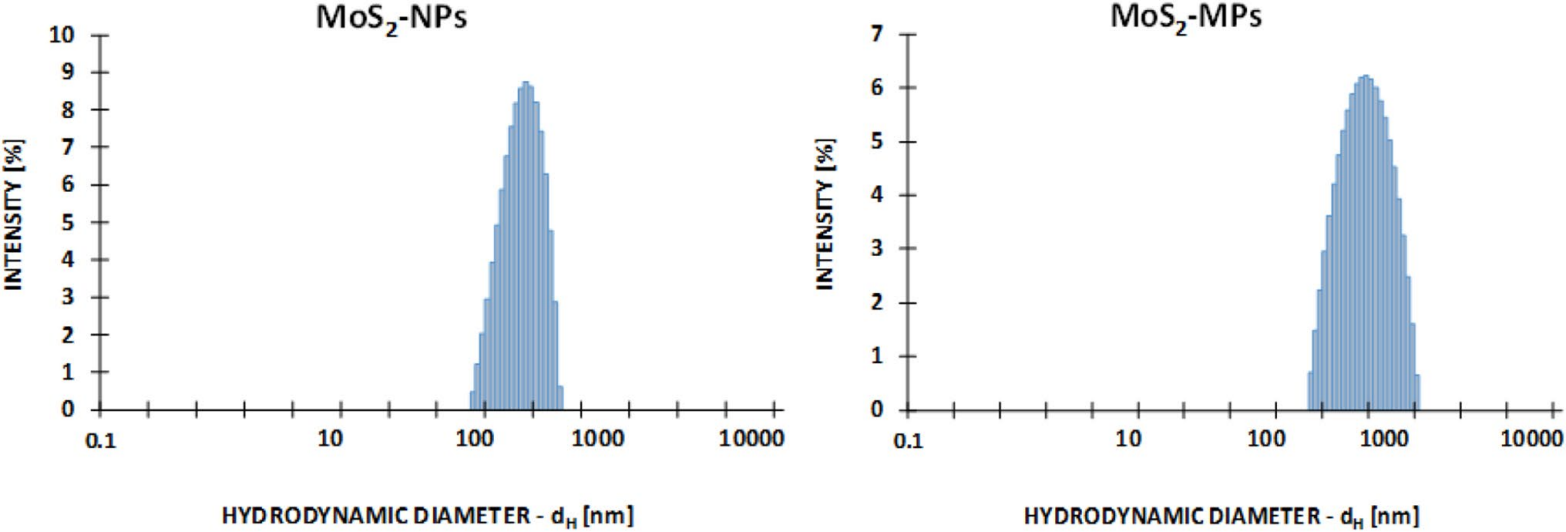
DLS histograms determining the hydrodynamic diameter of the MoS_2_ particles

A stable aqueous dispersion, a colloid of MoS_2_-NPs as well as a suspension of MoS_2_-MPs stabilized by PVP, with the weight ratio of MoS_2_:PVP 1:1; K 90, Mw = 360 000 (Fluka) were prepared. The size of the NPs ranged 50‒100 nm, and that of MPs 0.5‒5 μm. The procedure of MoS_2_ particles preparation was described by Sobańska et al. [15]. In this paper, the authors also presented the 3-dimensional morphology of particles analysis (Fig. 1), including the size, shape and possible agglomerations of MoS_2_ particles using high resolution scanning electron microscopy (FEI-Nova NanoSEM 450). In the same paper, a size distribution histogram was presented using the dynamic light scattering (DLS) technique with zeta potential measurements (Fig. 2). DLS measurements revealed that the hydrodynamic diameter of the MoS_2_ particles was: dH MoS_2_-NPs = 251 ± 94 nm for nanoparticles and dH MoS_2_-MPs = 0.7 ± 0.3 μm for microparticles. As described earlier MoS_2_-NPs in solution were dispersed and stabilized by PVP, so the high molecular weight polymer strongly increased the hydrodynamic diameter of the MoS_2_-NPs nanoparticles compared to the diameter measured by the HR-SEM technique. In the end, the internalization of MoS_2_-NPs as well as MoS_2_-MPs was performed by scanning transmission electron microscopy (STEM) with energy-dispersive X-ray spectroscopy (EDS) [15]. The volumes of the PVP solution, as well as of the solutions of the tested nano- and micro-MoS_2_, administered each time to the trachea of the rats, were calculated individually for each animal, maintaining the proportions depending on their body weight. More specifically, 100 µL of the substance was administered per 250 g of the rat’s body weight. The physico-chemical analysis of the prepared MoS_2_ suspensions indicates that they were nano- and micrometric forms with satisfactory stability to perform biological tests.

### Tissue sample preparation

The blood samples from the rats’ tail veins were collected twice into EDTA tubes (SARSTEDT), before and after intratracheal instillation. All blood samples were collected from the right ventricle of the heart into Monovette 7.5 mL containing lithium heparin (SARSTEDT) during the dissection. All blood samples were stored at −20 °C until analysis.

The soft tissue samples collected during autopsy were weighed (approx. 100 mg each) using the analytical balance Sartorius (BA 210S), and then they were mineralized under the appropriate conditions using an UltraWave mineralizer (Milestone, SpectroLab). The mineralization process was carried out according to the program using concentrated HNO_3_.

For determination and tissue distribution of Mo in the tissue samples using LA-ICP-MS analysis, the brain, spleen, lung and liver samples were first formalin-fixed and then paraffin-embedded (automated Belair RVG/1 Vacuum Tissue Processor; TES 99 Tissue Embedding System). Then, the paraffin blocks were cut into 20 µm thick sections (HM 325 Rotary Microtome). In order to check the analyte contamination, all solutions (formalin, paraffin, agarose and the PVP solution) were determined using the inductively coupled plasma excitation mass spectrometry (ICP-MS) technique.

The bronchoalveolar lavage cells and lung tissues were processed using routine histopathological protocols (light microscopy using Giemsa and hematoxylin-eosin staining; electron scanning microscopy with lead and osmium contrasting) to evaluate tissue changes and MoS_2_ particles distribution in the cells in different organ compartments.

### Instrumentation and experimental parameters

Molybdenum determination in blood samples was performed by ICP-MS (ELAN DRC-e, PerkinElmer, SCIEX, USA) using a dynamic reaction cell (DRC) with methane (CH_4_) reaction gas, eliminating spectral and matrix-derived interferences. The linear calibration curve for Mo determination in blood ranged 0.5‒50 μg L^−1^ with the correlation coefficient *r* = 0.9999. The analytical precision of the method amounted to 5.4%. The repeatability of measurements using the Seronorm™ Trace Elements Whole Blood as an internal quality control amounted to 8.8%. The limit of detection (LOD) based on 3*standard deviation (SD)/slope, by the 5 repetitive analysis of the response of the curve amounted to 0.026 µg L^−1^ and the limit of quantitation (LOQ: 6*SD/slope) amounted to 0.052 µg L^−1^.

The mineralized tissue samples were diluted appropriately before the analysis using deionized water. Molybdenum determination in the mineralized tissue samples was performed using the ICP-OES technique (Agilent 5100 SVDV, MS Spectrum). The ranges of the calibration method for Mo determination in the mineralized tissue samples ranged 2‒500 μg L^−1^ with the correlation coefficient *r* = 0.9999. The analytical precision of the method amounted to 1.6%. The repeatability of measurements using a solution of Mo with a concentration of 100 µg L^−1^ amounted to 2.7%. The LOD and LOQ estimated using 10 blank samples amounted to 0.16 µg L^−1^ and 0.32 µg L^−1^, respectively.

The bioimaging of tissue slices was performed by the LA system (J200 Tandem LA/LIBS Applied Spectra Inc., USA) with LA-ICP-MS used as a complementary analysis. The ranges of the calibration method for Mo determination in gel standards ranged 0.5‒50 μg L^–1^. An intra- and inter-assay coefficient of variability as well as the recovery of Mo per DOLT-5 pellet (8 replicate ablation lines) amounted to 12.9%, 16.5% and 90%, respectively. The LOD and LOQ estimated using 10 blank agarose samples amounted to 0.017 µg g^−1^ and 0.034 µg g^−1^, respectively.

### Statistical analysis

Quantitative data were presented as mean ± standard deviations (SDs). The mean and SD values were calculated using the GraphPad Prism Software v.6.01 for Windows (GraphPad Prism Software, Inc., USA). Due to a small number of observations per group, the data were assumed to lack normal distribution. Therefore, the Kruskal–Wallis test with Dunn’s post hoc test were used for determining statistical significance. The statistical significance was set at *p* < 0.05.

## Results

### Tissue content

#### Analysis of Mo in blood samples by ICP-MS

**Fig. 3:**
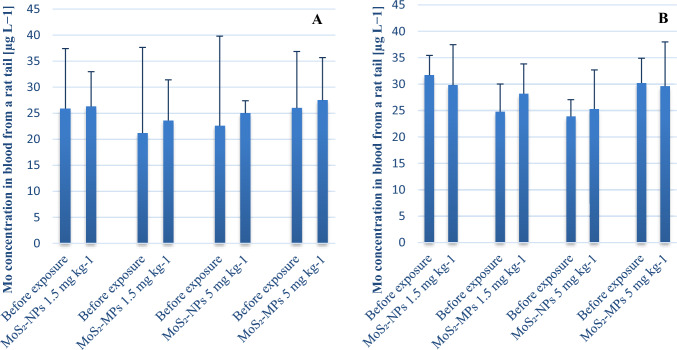
Mo concentration (mean ± SD) in the venous blood collected from the tail vein of male rats exposed to MoS_2_-NPs and MoS_2_-MPs after a single administration, and the analysis performed after 24 h (**a**) and 7 days (**b**), at a dose of 1.5 and 5 mg MoS_2_ per kg b.w. In individual groups, the same rat was bled before exposure, and after 24 h or 7 days. The study groups were composed of 3 animals

In the tail venous blood obtained from the animals before exposure, Mo concentrations ranged 10.2‒38.4 μg L^−1^ (mean: 22.4 ± 8.0 μg L^−1^, median: 22.2 μg L^−1^). Blood was collected from the same animal before and after exposure, i.e., changes in Mo concentrations were monitored in each animal individually. After 24 h of exposure, a slight increase in Mo concentration was observed; however, this increase was not dependent either on the form of MoS_2_ or its dose (Fig. 3a). In addition, there were no statistically significant differences between the groups. Similarly, there were no differences in Mo concentrations between the groups after 7 days of exposure (Fig. 3b).

**Fig. 4:**
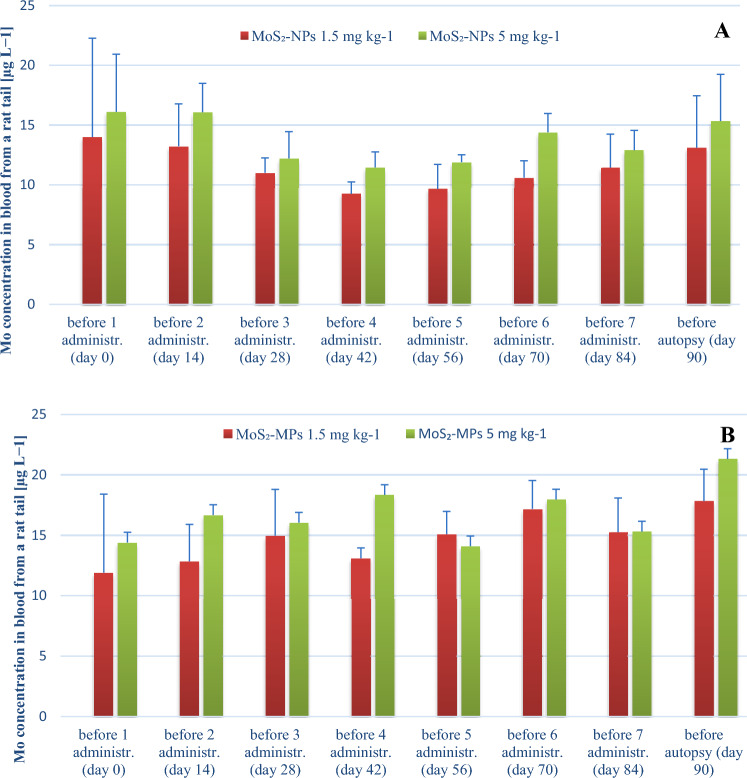
Mo concentration (mean ± SD) in the venous blood collected from the tail vein from male rats exposed to MoS_2_-NPs (**a**) and MoS_2_-MPs (**b**) after 7 administrations at 2-week intervals, and with the analysis performed over 90 days, at a dose of 1.5 and 5 mg MoS_2_ per kg b.w. within each group, the same rat was bled after exposure at specific time intervals. The study groups were composed of 3 animals

In the venous blood collected from the tail of animals after exposure for 7 times at 2-week intervals and with the analysis performed within 90 days, Mo concentrations were comparable to each other and they were not dependent either on the form of MoS_2_ or its dose (Fig. 4).

For MoS_2_-NPs (at both doses), a slight decrease in Mo concentration was observed from the first administration, which then slowly increased from the fourth administration. For MoS_2_-MPs (also at both doses), a slight increase in Mo concentration was observed from the first administration, which then slowly increased again from the fourth administration.

After the determination of Mo concentration in the venous blood taken from the right ventricle of the animals’ hearts during autopsy performed after 90 days of exposure to MoS_2_ at a higher dose (5 mg kg^−1^), an approximately twofold increase in Mo concentration for both forms was shown (a slightly higher concentration for MoS_2_-MPs: 15.3 ± 3.9 vs. 21.3 ± 7.3 μg L^−1^, respectively, for MoS_2_-NPs and MoS_2_-MPs. A similar relationship was observed in venous blood, in which the Mo concentration before autopsy after 90 days was slightly higher for MoS_2_-MPs (17.8 ± 2.6 μg L^−1^) than for MoS_2_-NPs (13.1 ± 4.3 μg L^−1^) at a dose of 1.5 mg MoS_2_ kg^−1^ b.w.

#### Kinetic profiles for MoS_*2*_-MPs and MoS_*2*_-NPs

**Fig. 5:**
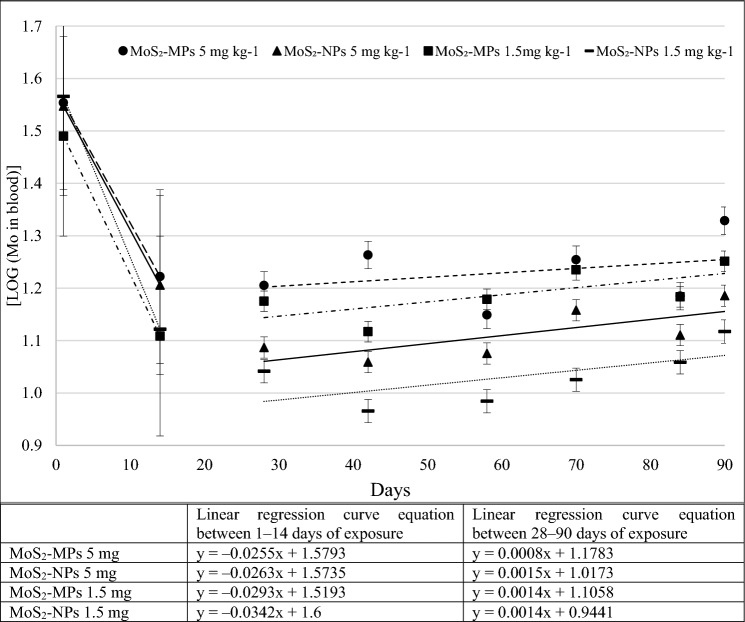
The mean blood concentration–time relationship for MoS_2_-MPs and MoS_2_-NPs after intratracheal instillation to rats. The logarithm linear regression curve equation. Each data point represents the mean and SD of Mo concentrations measured (*n* = 6)

The multiple-dose toxicokinetics in the experiment performed reflects how the body responds to substances introduced intratracheally. The concentration curves of various forms of Mo in the blood over time, after repeated administration of 2 different fixed doses (1.5 and 5 mg kg^−1^), are presented in Fig. 5.

The trend indicating the presence of 2 phases is visible. During the first phase, the Mo concentration in the blood decreases until day 14 (the Mo concentration before the second administration), below the pre-exposure concentration. The second phase is linear, less abrupt and practically flat, but with an increasing trend towards the end of the experiment. The multi-compartment model assumes an exponential curve of Mo concentration over time with different half-lives for the distribution and elimination phases of MoS_2_-MPs and MoS_2_-NPs. Following the intratracheal instillation, the distribution half-life was the fastest for the lower MoS_2_-NPs dose (1.5 mg kg^−1^) at *T*_1/2_ 8.8 days. At the same dose, the calculated *T*_1/2_ was 2 days longer for the MoS_2_-MPs. MoS_2_-NPs and MoS_2_-MPs at the higher dose (5 mg kg^−1^) showed similar values of *T*_1/2_ 11.5 vs. 11.8 days, respectively. The multiple-dose elimination trend is increasing for both formulations in the blood Mo concentration.

**Fig. 6:**
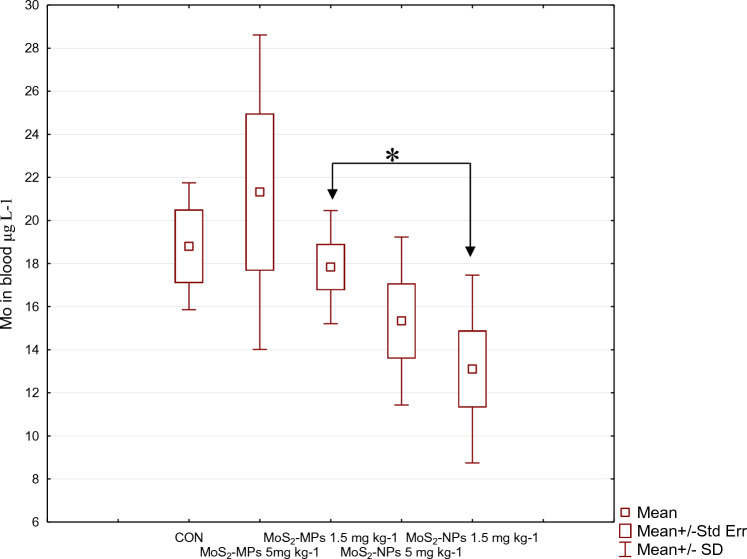
Median blood concentration for MoS_2_-MPs and MoS_2_-NPs for both doses (5 mg kg^−1^ and 1.5 mg kg^−1^) before autopsy (90 days)

Although the multiple-dose elimination trend is increasing for both formulations in the blood Mo concentration, compared to the control group at day 90, all concentrations (except MoS_2_-MPs at a dose of 5 mg kg^−1^) were lower (Fig. 6). MoS_2_-MPs were absorbed in higher amounts and was more slowly removed from the bloodstream. For MoS_2_-MPs it could be seen that after 90 days the concentrations were higher than at the beginning of the experiment, so it was excreted more slowly. After 90 days of the experiment, MoS_2_-NPs concentration returned to their baseline thus, excretion even after a repeated doses was fast.

Statistically, for MoS2-NPs and MoS2-MPs at the lower dose of (1.5 mg kg^−1^) the differences are statistically significant *p* = 0.046, and despite apparent differences there is no statistical significance for MoS_2_-NPs and MoS_2_-MPs at a higher dose (5 mg kg^−1^).

#### Analysis of Mo soft tissue samples by ICP-OES

##### Analysis of Mo concentration in the rat’s lung

**Fig. 7:**
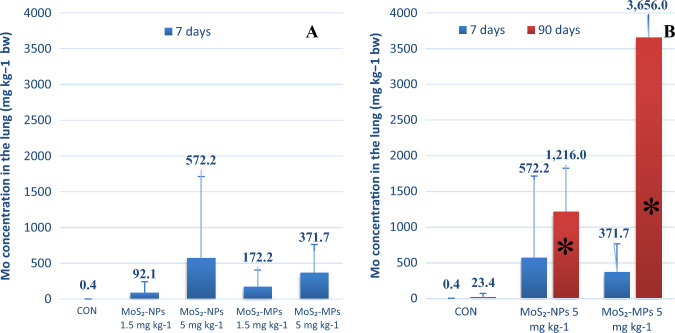
Molybdenum concentration (mean values ± SD) in the lungs of male rats exposed to MoS_2_-NPs and MoS_2_-MPs after: **a** single administration, and the analysis performed after 7 days, at a dose of 1.5 and 5 mg MoS_2_ per kg b.w., vs. CON (*N* = 3‒4); **b** 7 administrations (at 2-week intervals), and the analysis performed after 90 days, at a dose of 5 mg MoS_2_ per kg b.w., vs. CON (*N* = 4); The PVP was administered in a volume of 400 µL of PVP per kg b.w. (*N* = 4) (CON)

In accordance with macroscopic observations indicating an uneven distribution of Mo in the sampled tissues, the lung tissue collected during the autopsy was completely mineralized. Measurements of the Mo content in the lung tissue performed 7 days after exposure to MoS_2_ showed a large scatter of individual values (Fig. 7a). A greater accumulation of MoS_2_-MPs in the lung tissue was observed. The concentration of Mo in the lungs of animals repeatedly exposed to MoS_2_, both MoS_2_-NPs and MoS_2_-MPs, was significantly higher compared to a single administration of MoS_2_, proving the material accumulation of the tested preparations in the lungs of the exposed rats. The determination of Mo content in the lung tissue after exposure to MoS_2_ in 90 days showed and confirmed a several times higher accumulation for MoS_2_-MPs compared to MoS_2_-NPs (Fig. 7b). Differences between the results of Mo concentrations in individual rats may be due to the actual differences in the baseline Mo status, the animals’ natural adaptive abilities disorder and the Mo absorbed after administration.

##### Analysis of Mo concentration in the rat’s liver

The measurements of Mo content in the liver tissue performed 7 days after exposure to MoS_2_ showed no differences between the groups of exposed animals (there was no dependence on the administered dose) or in relation to the control group which was administered PVP (Table 2). The analysis of Mo content performed after 90 days showed a slightly higher concentration for MoS_2_-NPs compared to MoS_2_-MPs. However, these values did not differ significantly between each other or in relation to the control group which was administered PVP.

**Table 2:** Molybdenum concentration (mean values ± SD) in the liver and the spleen of male rats exposed to MoS_2_-NPs and MoS_2_-MPs after: single administration, and the analysis performed after 7 days, at a dose of 1.5 and 5 mg MoS_2_ per kg b.w., vs. CON (*N* = 4); 7 administrations (at 2-week intervals), and the analysis performed after 90 days, at a dose of 5 mg MoS_2_ per kg b.w., vs. CON (*N* = 4); PVP was administered in a volume of 400 µL PVP per kg b.w. (*N* = 4) (CON)

Organ	Parameter	Interval	
		7 days	90 days
Liver	CON	1.19 ± 0.49	1.09 ± 0.34
	MoS_2_-NPs 1.5 mg kg^−1^	1.10 ± 0.49	–
	MoS_2_-NPs 5 mg kg^−1^	1.24 ± 0.61	1.67 ± 0.38
	MoS_2_-MPs 1.5 mg kg^−1^	1.28 ± 0.51	–
	MoS_2_-MPs 5 mg kg^−1^	1.09 ± 0.45	1.38 ± 0.23
Spleen	CON	0.15 ± 0.02	0.16 ± 0.06
	MoS_2_-NPs 1.5 mg kg^−1^	0.27 ± 0.15	–
	MoS_2_-NPs 5 mg kg^−1^	0.16 ± 0.05	0.87 ± 0.09
	MoS_2_-MPs 1.5 mg kg^−1^	0.14 ± 0.10	–
	MoS_2_-MPs 5 mg kg^−1^	0.16 ± 0.05	0.32 ± 0.08

##### Analysis of Mo concentration in the rat spleen

The measurements of Mo content in the spleen tissue performed 7 days after exposure to MoS_2_ showed no differences between the groups of exposed animals (there was no dependence on the administered dose) or in relation to the control group which was administered PVP (Table 2). Similar to the liver tissue, the analysis of Mo content performed after 90 days showed a higher concentration for MoS_2_-NPs compared to MoS_2_-MPs. However, these values did not differ significantly between each other or in relation to the control group which was administered PVP.

##### Analysis of Mo concentration in the rat’s brain

The results for the brain were below the calculated detection limit, indicating that Mo concentrations were at trace levels.

### Tissue distribution

#### Analysis of Mo in soft tissue samples by LA-ICP-MS

**Fig. 8:**
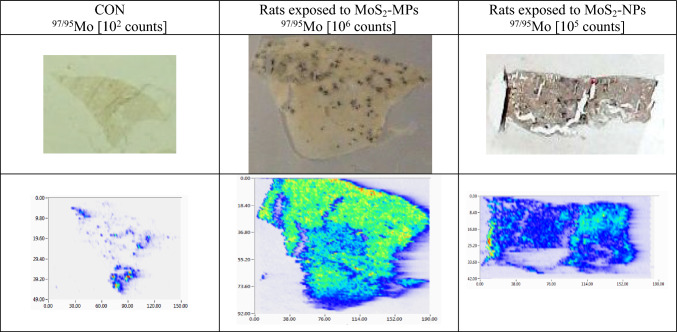
LA-ICP-MS Mo bioimaging Mo (the ^97/95^Mo ratio) in the lung sections of the rats exposed to MoS_2_-NPs and MoS_2_-MPs vs. CON, normalized to the size of the sample

There are few studies using laser bioimaging techniques that can be very helpful in assessing the distribution and concentration of NPs in tissues as an important tool in assessing toxicity. Taking into consideration the complexity and multidirectional nature of factors determining the toxicity of NPs, biological studies should be carried out in many directions. Direct micro-sampling of solids allows for determining distribution, i.e., for obtaining detailed images of specific tissue regions of the selected elements on the surface of a solid sample (mapping). In this paper, the tissue sections from soft tissues (brain, lung, liver, spleen) were used for the confirmation of tissue distribution of both Mo forms using the LA-ICP-MS technique as a complementary tool in the experiment. Figure 8 shows an example of tissue distribution for Mo. The higher signal intensity for the MoS_2_-MPs in the lung tissue analyzed after 90 days at a dose of 5 mg MoS_2_ per kg b.w. confirms the results obtained by ICP-OES. Moreover, an analysis of Mo distribution in the liver and spleen samples revealed a higher concentration of MoS_2_-NPs in the same dose of 5 mg MoS_2_ per kg b.w. after 7 administrations, which was in accordance with the results obtained by ICP-OES. The bioimaging analysis confirmed the different tissue distribution of Mo and its non-heterogeneity. The concentration of Mo in the brain tissue was the lowest compared to other tissues and was below the calculated value of LOD (0.017 µg g^−1^) for LA-ICP-MS, which was also in good agreement with ICP-OES data.

#### Analysis of MoS_2_ particle distribution in BAL cells and lung tissue

**Fig. 9:**
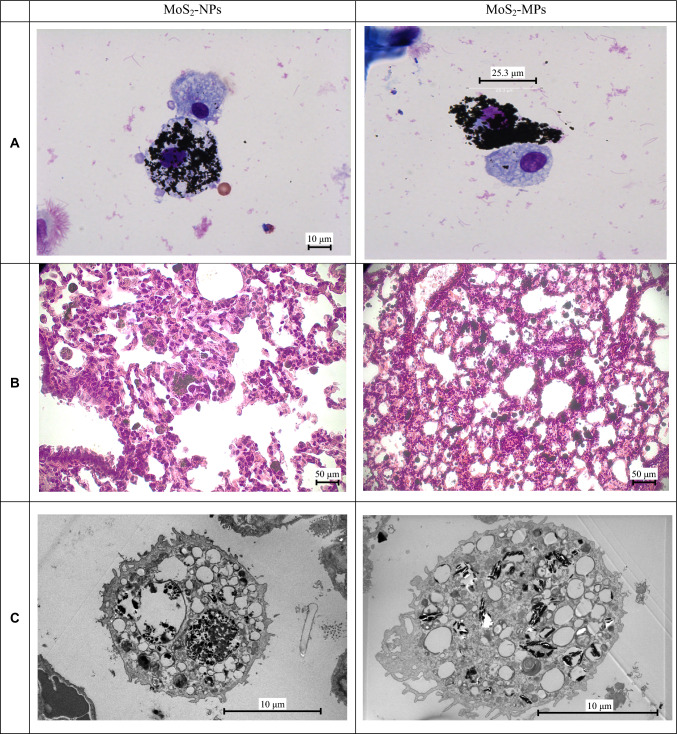
Exemplary images of the lung tissue of the rats exposed to 5 mg kg^−1^ bw and analysed after 7 days. **a** Alveolar macrophages isolated from BAL heavily loaded with MoS_2_ particles (Giemsa staining). The cells attached to the macrophages are probably monocytes. **b** Light microscopy images of the lung tissue showing MoS_2_ particles (dark brown or black agglomerates) present in alveolar macrophages and interstitial tissue (routing HE staining 200×). **c** SEM pictures of macrophages loaded with MoS_2_ particles. The particles are engulfed in clusters in vesicular organelles (endosomes)

Exemplary images of the alveolar macrophages isolated from bronchoalveolar lavage (BAL) and the lung tissues of the rats exposed to 5 mg kg^−1^ b.w. and analysed after 7 days are shown in Fig. 9. Based on the images an efficient internalization of MoS_2_ particles by the alveolar macrophages can be proven, without induction of visible morphological changes of the macrophages as well as the cells in the interstitial tissue. The similar changes indicating an efficient clearance of the particles without induction of profibrotic reaction could be observed in the animals at the end of the experiment, i.e., after 90 days of exposure.

## Discussion

### Toxicokinetics parameters of MoS_***2***_ in both forms in the rats

#### Inhalatory route

The rate of absorption of Mo depends, inter alia, on its solubility. In contrast to MoO_3_, MoS_2_ particles are practically insoluble in water, hence their absorption from the lung tissue is expected to proceed at a very slow rate [21, 22]. Quantitative estimates of absorption following inhalation exposure to molybdenum in humans or animals were not identified [23]. Some evidence for absorption of molybdenum trioxide from the airways mucosa is available from inhalation studies on molybdenum trioxide conducted in rodents, i.e. in guinea pigs [24] well as rats and mice [25]. To our knowledge, our study is the only one available in the published literature which evaluated kinetics parameters in rats exposed via inhalatory route to MoS_2_ particles, hence any reliable comparisons to the published data cannot be made.

#### Oral and intravenous routes

Available data on Mo kinetic parameters after exposure via other routes indicate its rather fast absorption, distribution and elimination, depending to a great extent on the chemical form of Mo. In the study conducted by Werner et al. [26] on volunteers, the elimination of Mo from the blood after a single intravenous injection of a trace quantity of Mo (ranging 300‒450 μg) occurred in the *T*_1/2_ range of 4‒70 min (half the time of the fast component of clearance) and in the *T*_1/2_ range of 3‒30 h (half the time of the slow component of clearance) in a two-compartment model. In the presented biokinetic model, the authors claimed that the clearance of plasma was much faster than the literature data. In addition, the authors showed that the volumes of distribution were significantly higher than the plasma volumes, but smaller than the calculated extracellular spaces. The authors further claimed that the faster Mo clearance from plasma might be explained by a quick uptake of Mo into tissues. This may indicate a very fast distribution of Mo in body fluids. The slight differences in results observed in our paper may be explained by both physiological inter-individual differences and also by the sampling schedule. Moreover, we also assume it may be related to the changing physiology of animals with maturation leading to lower demand for Mo in a growing organisms and eventually lower Mo plasma concentration. Unfortunately, we were not able to identify studies which could provide any support for such hypothesis (and as mentioned above no studies were found on relevant Mo blood kinetic parameters after inhalation exposure to particulate forms of Mo). It can be assumed that an unknown fraction of each dose administered after various time intervals was absorbed from the lungs (not known if in the form of particles or ions released after dissolution of the particles), causing comparable spikes in Mo concentration, but it was afterwards efficiently eliminated from the blood before the next dose. It is probable that within 2 weeks between administrations an equilibrium of the Mo distribution between the tissues and the blood has been established, which did not lead to increased Mo blood concentrations. As we calculated, the expected elimination half-life was *T*_1/2_ 354 vs. 195 days for the higher dose (5 mg kg^−1^) of MoS_2_-MPs vs. MoS_2_-NPs, respectively, compared with an elimination half-life of *T*_1/2_ 221 vs. 212 days for the lower dose (1.5 mg kg^−1^) of MoS_2_-MPs vs. MoS_2_-NPs, respectively. Based on these data, we hypothesize that, after repeated dosing, there is quite a rapid absorption of a certain amount (most probably very small) of the particles fraction administered (including ions after dissolution), while the remaining part of the particle fraction accumulates in the lungs. Moreover, For MoS_2_-MPs it could be seen that after 90 days the concentrations were higher than at the beginning of the experiment, so it was excreted more slowly. This is consistent with the observations of Kuraś et al. [27], as a lot of MoS2-MPs are observed in the lungs, the same MoS2-MPs absorption into the blood is faster, greater and excretion longer, as confirmed in Fig. 6.

Turnlund and Keyes [28] conducted a study on the clearance of Mo from the blood in men after administration of Mo, first intravenously (33 μg of ^97^Mo) and then orally (22 μg day^−1^ Mo). The administration of Mo increased both the natural intrinsic Mo in plasma and the total Mo in plasma during the first minutes (6.9 vs. 6.9 nmol L^−1^, respectively) up to 1 h (13.0 nmol L^−1^ vs. 17.1 nmol L^−1^, respectively), then it again decreased to near baseline after 24 h of uptake (5.7 nmol L^−1^ vs. 6.0 nmol L^−1^, respectively). Eventually, 48 h after infusion Mo concentration remained at a similar but only slightly lower level (5.1 mmol L^−1^ vs. 5.3 mmol L^−1^, respectively). After 72 days, Mo concentration remained unchanged (5.8 ± 2.5 nmol L^−1^). These findings on urinary excretion are in agreement with the data obtained by Werner et al. (2000). The authors of the study suggest that the introduced Mo disturbed the overall Mo metabolism at the beginning of the experiment. It resulted in an increased level of Mo after exposure, combined with the physiological level of natural Mo. More specifically, Mo could have been absorbed by the tissues that released the pool of bioavailable intrinsic Mo in the body increasing its concentration in the blood. Further exposure to low dietary Mo may have resulted in physiological adaptation [28]. Another study concerning compartmental modeling to explain the alteration in Mo distribution and excretion with the urine showed a positive correlation in the studied men, where increased Mo intake was associated with both increased Mo absorption and urinary excretion. The fraction deposited in tissues was inversely correlated [29]. It is known that Mo is mainly excreted in the urine and it is a key pathway for modulating exposure to Mo in the body. Molybdenum from feces is eliminated in lower amounts. In humans, it is up to 17‒80% of the total absorbed Mo dose [30, 31], but Giussani et al. [32] and Novotny and Turnlund [29] reported that this excretion was on the level of 75–90% of the absorbed Mo dose. Urinary Mo excretion, according to the results obtained by Bell et al. [33] after oral administration to rats, showed that 90% of the dose was eliminated by the kidneys. The lack of multiple urine collection from the freely moving rats may be considered a limitation of this article. This was not included in the study implementation schedule because attention was focused on the intratracheal instillation exposure and on following the Mo metabolism and key pathways of its regulation connected with blood kinetics and tissue distribution.

#### Induction of pro-inflammatory reactions in the lung

The latest research has revealed that MoS_2_ has the ability to cause inflammatory reactions [31, 34]. It was shown that MoS_2_-MPs as well as MoS_2_-NPs deposited in the lung tissue of the rats after intratracheal instillation may cause inflammatory reactions, although a stronger response was observed for MoS_2_-MPs. The authors observed inflammation in the respiratory system in the rats after a single administration. The difference in the inflammatory response was statistically significant for both doses (1.5 and 5 mg MoS_2_ kg^−1^ b.w.) 7 days after the autopsy for MoS_2_-MPs compared to control (PVP) rats [15]. Moreover, the authors showed interstitial inflammation at a higher dose, both 24 h after the autopsy (for both forms) and 7 days after the autopsy for MoS_2_-MPs. This data is confirmed by the results presented in our paper. STEM with EDS unambiguously revealed multiple alveolar macrophages loaded with plate-shaped Mo-MPs as well as agglomerates of Mo-NPs. The characteristically expanded lysosomes in these macrophages containing similar clusters of particles were observed in the cytoplasm of macrophages. The authors also showed the presence of NPs in epithelial cells, which may suggest that the process of internalization indicates the possibility of NPs penetration through the epithelium and systemic circulation extended clearance [15]. Chng et al. [35] noticed that disk-shaped particles were conducive to proinflammatory reactions in the respiratory system. Moreover, the histopathological assessment after chronic inhalation of 6.6 mg MoO_3_ mg/m^3^ in mice revealed significantly greater instances of adenoma or carcinoma of alveolar/bronchiolar in the exposed groups in comparison to control ones [36]. Furthermore, in the same study the authors pointed to marginally greater incidents of lung tumor in male rats. The initial histopathological lung damages were observed already at 10 mg/m^3^. In another study, Huber and Cerreta [37] reported an increase in the neutrophils and multinucleated macrophages in BAL fluid in hamsters after one day of inhalation of 5 mg Mo/m^3^, and lymphocytes after 7 days of exposure. The increase in neutrophils in BAL fluid was also observed in mice after inhalation of 90 mg Mo/m^3^ [16]. What is more, the tidal volume was already decreased after the lowest exposure level (8 mg MoS_2_/m^3^). Another study conducted by Peña et al. [17] also confirmed a lung inflammation caused by MoS_2_ nanosheets after a single inhalation in mice. Inflammatory cytokines and extracellular vesicles as well as immune cells detected in BAL fluid effected on inflammatory status.

Another study assessed the toxicity of Mo-NPs on rat BRL3A, i.e., rat liver cells, after 24-h exposure [38]. The authors of this study observed a significant increase in the lactate dehydrogenase enzyme release at the Mo-NPs concentration of 250 μg m L^−1^—much higher than in the study conducted by Braydich-Stolle et al. [39]. Also, an increase in mitochondrial activity reduction occurred at a much higher concentration—250 μg m L^−1^ [38]. Mo supplementation significantly increased the activity of xanthine dehydrogenase/xanthine oxidase, sulfite oxidase and superoxide dismutase in the liver [40]. This is also confirmed by a study conducted by Yang and Yang [41]. The authors investigated an effect of Mo supplementation (0.1 mg Mo L^−1^) of rats on the concentration of hepatic Mo, which was increased significantly compared to controls. Thus, it is very likely that the increased concentration of Mo in this study, observed after 7 administrations, caused disturbances in the metabolism of liver enzymes due to tissue retention.

Moreover, Mo is an essential trace element, which, as an enzyme component, supports iron metabolism and thus contributes to hematopoesis. Accumulation of Mo in tissues can cause the risk of anemia [42, 43]. In our study, we observed deviations in basic hematological parameters in exposed animals. Similar to Sobańska et al. [15] study. Therefore, lower Mo concentrations in blood after exposure are associated with hematological changes and damage to the vascular system during material collection, that directly affects hematological parameters (decrease in red blood cells, lower Mo concentrations). Kusum et al. [44] obtained similar results. According to authors, oral exposure to Mo in goats may altered haematological profile, because it causes a state of secondary copper deficiency. As a consequence, the study revealed significant reduce in mean hemoglobin, packed cell volume, total leukocyte as well as erythrocyte count. The mean of corpuscular hemoglobin concentration was also significantly decreased. Lyubimov et al. [45] confirmed a decrease in erythrocyte count as well as hematocrit in rats after gavage administered by 4.4 mg Mo/kg/day. Moreover, in the study Asadi et al. [46] the number of white blood cells increased with increasing levels in Mo NP dosage, after intraperitoneal injections in rats. NPs cause inflammation due to disorders in the lymphatic system.

In the described study, rats were exposed by intratracheal administration to nano- and micro-metric forms of Mo. It can be concluded that, after such exposure, MoS_2_-NPs as well as MoS_2_-MPs were mostly retained in the lung tissues. Distribution of the administered molybdenum disulfide particles was also observed in extrapulmonary tissues. Repeated exposure resulted in a significant accumulation of particles in both lungs and other tissues, with the following order of concentration: liver > spleen > brain. The distribution exponent was the fastest for the lower nanoparticle dose at *T*_1/2_ 8.8 days. The calculated elimination half-life was also faster for the nano-forms of Mo in comparison to the micro-forms, regardless of the dose.

The present results provide a solid basis for further research on the fate of nanoparticles in the body. Additional studies, such as information on the extent of oral exposure after inhalation exposure, are necessary to clarify the routes of exposure. In addition, this is the first study in which 3 techniques were used to complement each other for the evaluation of the effects of intratracheal instillation of MoS_2_-MPs and MoS_2_-NPs on tissue distribution in rats. The LA-ICP-MS technique was proposed as a complementary tool for ICP-OES and ICP-MS, for the identification as well as bioimaging of different sizes of Mo particles in rat tissue. The impact of the particle size and form was investigated, which may be an important tool in further internal biokinetics studies. Intratracheal exposure to Mo particles showed their retention and deposition, mainly in the lung tissue, in the form of MoS_2_-MPs, and to a lower extent in the liver and spleen, but mainly in the form of MoS_2_-NPs. Taking into consideration the complex nature of factors determining the toxicity of NPs and MPs, biological as well as toxicological studies should be carried out multidirectionally.

## Data Availability

Data generated and/or analyzed during the current study are available from the corresponding authors upon reasonable request.
